# Translation of the Chinese version of the modified Yale Food Addiction Scale 2.0 and its validation among college students

**DOI:** 10.1186/s40337-021-00471-z

**Published:** 2021-09-16

**Authors:** Hui Zhang, Tong Tong, Ye Gao, Chunguang Liang, Haitao Yu, Sisi Li, Xiangru Yan, Liying Wang

**Affiliations:** School of Nursing, Jinzhou Medical University, Jinzhou, 121000 China

**Keywords:** Food addiction, Psychometric properties, Validation, Yale Food Addiction Scale, Impulsivity, Self-esteem, Eating behaviour, Factor analysis

## Abstract

**Background:**

Obesity prevalence has substantially increased in China over the past decade. In China, over 1 in 7 individuals meet the criteria for overall obesity, and 1 in 3 meet the criteria for abdominal obesity, obesity has become a significant problem. Studies have shown that food addiction and obesity are inextricably linked. The modified Yale Food Addiction Scale 2.0 (mYFAS 2.0) is a brief measurement for assessing food addiction. This study aimed to explore the structure of the Chinese version of the mYFAS 2.0 and assess the occurrence of food addiction in a sample of college students in Northeast China.

**Methods:**

A cross-sectional design was conducted in a sample of 1099 undergraduate students in Northeast China. Participants completed the sociodemographic questionnaire, the Chinese version of the mYFAS 2.0, the Barratt Impulsiveness Scale (BIS-8), and the Self-Esteem Scale (SES) to test the hypothesis. Exploratory factor analysis and confirmatory factor analysis were performed to examine the underlying factor structure of the mYFAS 2.0. Two weeks later, 62 students who participated in the first test were recruited to evaluate the test–retest reliability.

**Results:**

The Chinese version of the mYFAS 2.0 demonstrated adequate internal consistency, good test–retest reliability and satisfactory construct validity. The results of the confirmatory factor analysis found that the Chinese version of the mYFAS 2.0 demonstrated a good fit to the two-factor solution identified by the exploratory factor analysis and showed superior fit indices compared to the one-factor model. The prevalence of food addiction in our sample was found to be in line with rates observed in other Asian and Western samples. The mYFAS 2.0 symptom count scores were correlated with BMI, the idea of dieting to lose weight, the desire to overeat, low self-esteem, and impulsivity.

**Conclusion:**

The results indicate that the Chinese version of the mYFAS 2.0 has good reliability and validity, and that it can be considered a tool to evaluate the addictive eating behaviours of undergraduate students.

**Supplementary Information:**

The online version contains supplementary material available at 10.1186/s40337-021-00471-z.

## Plain English summary

This study examines the construct validity of the Chinese version of the mYFAS 2.0 and explores the relationships between food addiction and self-esteem, impulsivity, and other clinical variables. The results show that the Chinese mYFAS 2.0 scale has a two-factor structural solution and has good psychometric characteristics. Of 1099 college students in Northeast China, the rate of food addiction was 6.7%. In addition, food addiction scores are associated with BMI, the idea of dieting to lose weight, the desire to overeat, self-esteem, and impulsivity.

**Supplementary Information:**

The online version contains supplementary material available at 10.1186/s40337-021-00471-z.

## Background

Food addiction reflects a substance use disorder (SUD) framework [1], which refers to the transformation of the primary self-balance regulation mechanism of food intake into a hedonic regulation mechanism [2]. Evidence is emerging that certain foods, especially those high in refined sugars and fats, may be capable of triggering an addiction-like eating response in vulnerable individuals [3]. Such foods are the same as addictive drugs and alcohol because they can interfere with the reward mechanism of the brain's limbic system [4].

Food addiction, as a dysfunctional eating pattern, is usually associated with obesity and eating disorders (EDs) [5, 6]. Obesity is a global epidemic metabolic disease, and the increasing obesity rate in recent years has made obesity a global health issue [7]. In China, the prevalence of obesity rose from 3.1% in 2004 to 8.1% in 2018. In 2018, an estimated 85 million adults (48 million men and 37 million women) were obese, three times the number of adults with obesity in 2004 [8]. However, unlike other behavioural addictions or EDs, food addiction was not officially included in the fifth edition of the Diagnostic and Statistical Manual of Mental Disorders (DSM-5) or the International Classification of Diseases (ICD-11). The validity and applicability of the food addiction structure have been controversial [9, 10]. For example, food is necessary for human survival, and food addiction requires distinction from the physiological need to ingest sufficient calories to maintain body weight; some researchers believe that the concept of food addiction oversimplifies complex behavioural phenomena [11]. An argument often used against the notion of food-directed use disorders is the difficulty of identifying which ingredient in the food is responsible. It is not yet clear which macronutrients or combinations of macronutrients may cause food addiction, making it challenging to classify food abuse [12]. In addition, most research on the neurobiological mechanisms behind food addiction focuses on animal models but is rarely conducted with humans [13]. However, in recent years, the research on food addiction has significantly increased, arousing the interest of the scientific community in the correct classification and construction of food addiction [14–16]. Increasing research has supported the inclusion of food addiction in psychiatry [17]. For example, over the past 50 years, the modern food environment has been dominated by highly processed (HP) foods [18]. HP foods are as reinforcing as substances are in substance use disorders, and HP foods are more effective in activating the reward-related nervous system than minimally processed foods [19, 20]. The addictive nature of HP foods plays a crucial role in driving addictive diets [21]. Evidence shows that sugar is addictive, toxic and unrelated to calories [22, 23]. Chocolate can activate similar brain regions and neurobiological substrates and has a potential psychoactive effect similar to that of abused substances and increasing the sugar content of chocolate can enhance its role in mental function [24]. The study by Kevin and Jeremiah et al. used the addiction syndrome model as a guiding theoretical framework to examine the structure of food addiction, and found that people with food addiction had significant clinical dysfunctions in the 3 broad areas of cognition, emotion regulation, and behaviour, which provides support for the clinical significance of food addiction [25]. A recent study showed that food addiction reflects changes in brain–gut–microbiome (BGM) interactions. Cheap, delicious, high-calorie foods transform the homeostasis balance both inside and outside the gut to a hedonistic mechanism through central and intestinal mechanisms [2]. The systems biology model of BGM interactions proposed by Arpana et al. provides not only a reasonable explanation for many hard-to-cure obesity symptoms but also a theoretical basis for new treatment strategies [26, 27].

Currently, the Yale Food Addiction Scales are the sole existing tools used to assess food addiction [28, 29]. The YFAS was developed in 2009 based on the Diagnostic and Statistical Manual of Mental Disorders 4th edition Text Revision (DSM-IV-TR) diagnostic criteria for substance dependence [30]. The YFAS is a 25-item self-report measurement used to assess addiction to highly palatable foods (e.g., chocolate, ice cream, and pizza). The YFAS has been validated in several languages and cultures and has demonstrated adequate reliability and validity [31]. In 2014, the short version of the YFAS was developed. The modified YFAS (mYFAS) contained fewer questions than the original YFAS and was used to reduce the burden on participants in the screening process, and it has been shown to have similar psychometric properties to the YFAS [32]. The mYFAS is considered a suitable alternative for the full measure [31].

To reflect the changes in the diagnostic criteria of SUD in substance-related and addiction disorders in the DSM-5 released in 2013 (e.g., the addition of craving, the merging of abuse and dependence criteria, and the use of a diagnostic continuum of severity) [33], the YFAS 2.0 was developed in 2016 and consists of 35 items designed to capture 11 food addiction symptoms [34]. The YFAS 2.0 and the initial YFAS showed similar psychometric properties and estimated a similar prevalence of food addiction [29].

Schulte and Gearhardt developed a modified YFAS 2.0 (mYFAS 2.0) in 2017. The mYFAS 2.0 consists of 13 items (one item for each symptom and two items for clinically significant impairment or distress) [35]. The mYFAS 2.0 was validated as an abbreviated tool for large-scale epidemiological studies or a simple food addiction screening measure. Over the past five years, the mYFAS 2.0 has been translated into multiple languages [36–40], and it is widely used in Western countries. Currently, the mYFAS 2.0 has been validated in Brazil [36], Italy [37], the Czech Republic [38], France [39], and most recently, China [40]. Studies with clinical and non-clinical samples show similar validity indices, with proven reliability in each version. In 2021, Li Shaojie et al. translated the English version of the mYFAS 2.0 and verified the applicability of the scale in China. Li Shaojie et al. only evaluated the factor structure of the mYFAS 2.0 and did not perform exploratory factor analysis (EFA), and they used confirmatory factor analysis (CFA) to evaluate the fit of the originally proposed one-factor model [40]. The factor structure of different versions of the Yale Food Addiction Scale may be different in different countries. For example, the Malay version of the YFAS 2.0 comprises two factors: the psychological and the social dimensions, showing the Malaysian dichotomy of food addiction [41]. EFA is an important step that can be useful for refining measures, evaluating construct validity, and testing hypotheses [42]. EFA based on Chinese samples can verify the applicability of the mYFAS 2.0 with the Chinese cultural background, which can make the scale both scientific and reasonable and make the results more reliable. Whether the mYFAS 2.0 can be used directly to evaluate the degree of food addiction among college students in Northeast China needs to be confirmed.

Food addiction is strongly associated with disordered eating behaviours, experiences of food cravings, binge eating symptoms, and frequency of binge eating episodes [41, 43], and there is a significant positive relationship with mental health symptoms [19]. Furthermore, some studies have reported that food addiction is associated with a range of mental disorders, such as depression, anxiety disorders, posttraumatic stress disorders, and attention deficit hyperactivity disorders [44–46]. Therefore, evaluating the psychometric properties of the mYFAS 2.0 is considered an important topic in international food addiction research [44].

This study aimed to translate the original mYFAS 2.0 into simplified Chinese and further confirm its reliability and validity among college students in Northeast China. Furthermore, this study explored the connection between food addiction, clinical variables (e.g., dietary restraint and binge eating per week), sociodemographic characteristics, self-esteem, and impulsiveness.

## Methods

### Design and participants

A cross-sectional survey was conducted in Liaoning Province, China, from August to October 2020. Participants were college students from the cities of Shenyang and Jinzhou. All students provided informed consent before participating in the study. The research procedures complied with the ethical standards of the Ethics Committee of Jinzhou Medical College, as well as the 1964 Helsinki declaration and its later amendments.

This study was conducted with a convenience sample of undergraduate students from three universities in Liaoning Province (two of them are medical universities, and one is a comprehensive university). A total of 1173 students took part in the survey. During the survey, each class teacher assisted in the classroom. The authors and teachers explained the study's purpose and methods to the students. The questionnaires were individually delivered to each participant and completed in the presence of the authors and the teacher. The participants were encouraged to give truthful answers. Subjects who had not fully completed the mYFAS 2.0 and provided questionnaires with obvious logical errors were excluded (height was a significant outlier, and the number of binge eating times per week was greater than 50). We retained the remaining 1099 students (93.7%) as the subjects. The survey was anonymous except that two classes students were required to write their student numbers as the test–retest participants. Two weeks later, 62 students who participated in the first test were recruited to evaluate the test–retest reliability. All participants were native Mandarin speakers.

### Translation process

We obtained permission from Drs. Schulte to translate and verify the Chinese version of the mYFAS 2.0. We followed the systematic flow of Brislin's translation [47]. The mYFAS 2.0 was independently translated into Chinese by two medical doctors who are proficient in English. Then, together with the researchers, they compared the two Chinese versions of the questionnaires they had translated, discussed and corrected the inconsistencies and obtained the first draft of the Chinese version. According to Brislin's translation-back translation method [47], two English experts who had not been exposed to the scale translated the Chinese version of the first draft back into English. Finally, the original scale, the first draft of the Chinese version, and the translated English scale were compared and discussed by a psychologist and an expert familiar with Chinese and Western cultural nursing science to ensure that the semantics, standards, and concepts were as similar as possible, making the content of the scale more in line with the Chinese culture and language habits. Considering the different food preferences between China and the West, we replaced some food examples in the introduction of the scale. Finally, the pilot study was carried out among 10 medical students. They were invited to complete the scale and then asked about their understanding of the scale's introduction section, items, and options. We communicated with the survey respondents, and they reported that they had no difficulty understanding the content of each item of the scale, and the final Chinese version of the scale was obtained (for the final Chinese version of the mYFAS 2.0, see Additional File 1).

### Measurements

All participants completed the mYFAS 2.0 [35], the Self-Esteem Scale (SES) [48], and the Barratt Impulsiveness Scale Brief Version (BIS-8) [49]. Furthermore, participants were also asked to complete a checklist assessing sociodemographic variables (e.g., sex, age, and grade) and clinical variables (e.g., tobacco and alcohol use in the last six months, dieting ideas for weight loss, and the desire to overeat). Height and weight were also self-reported to calculate the body mass index (BMI) of each participant. Participants were categorized as underweight (< 18.5 kg/m^2^), normal weight (18.5–23.9 kg/m^2^), overweight (24–27.9 kg/m^2^), and obese (≥ 28 kg/m^2^) based on Chinese criteria of weight for adults [50].

### Modified Yale Food Addiction Scale 2.0

The mYFAS 2.0 consists of 13 questions: 11 items assess symptoms of food addiction, and two items assess diet-related impairment and distress. The scale is evaluated on an 8-point scale, ranging from 0 (never) to 7 (every day). It measures the experience of addictive eating behaviours during the past 12 months. Each item is scored dichotomously according to the threshold determined by the mYFAS 2.0 validation paper (0 = did not meet criterion, 1 = met criterion). This criterion is supported if any item corresponds to the diagnostic criteria, or the clinical severity meets the clinical threshold [35]. The cut-off values (i.e., which scores are coded with 0 and which scores are coded with 1) are shown in Additional File 2.

There are two scoring options for the mYFAS 2.0; one is a symptom count version (scores ranging from 0 to 11), which sum the diagnostic criteria that the subject meets. The other, Food addiction diagnosis, is a categorical diagnostic method which requires the presence of impairment/distress criteria. Hence, in assessing the diagnosis of food addiction, both the symptom count score and the clinical significance criterion were used (mild = 2–3 symptoms plus impairment or distress, moderate = 4–5 symptoms plus impairment or distress, severe = 6 or more symptoms plus impairment or distress).

### Desire to overeat

We asked about the frequency of desires to overeat with a single question: "How many times per week did you feel you wanted to eat more even after eating quite a lot of food throughout the last two hours?" The subjects filled in the number of times [51].

### The Self-Esteem Scale (SES)

The SES is a unidimensional measure of the global feeling of self-worth. The scale comprises ten items that are scored using a 4-point Likert format (from 1 = "strongly disagree" to 4 = "strongly agree"). The higher the score, the higher the degree of self-esteem. Questions 1, 2, 4, 6, 7, and 8 were positively scored questions, and questions 3, 5, 9, and 10 were negatively scored questions. The value of Cronbach's α was 0.77, and the test–retest reliability was 0.85 [48]. The Cronbach's α of the SES in this study was 0.83.

### The Barratt Impulsiveness Scale Brief Version (BIS-8)

The BIS-8 is designed to be a unidimensional impulsiveness measure, with higher scores reflecting higher impulsivity. The scale comprises eight items that are scored using a 4-point Likert format (1 = “rarely/never”, 2 = “occasionally”, 3 = “often”, 4 = “almost always/always"). Questions 1, 4, 5, and 6 were positively scored questions, and questions 2, 3, 7, and 8 were negatively scored questions. The Chinese version of the BIS-8 was validated in a sample of Chinese male prisoners and showed good reliability and construct validity [49]. In this study, the Cronbach's α of the BIS-8 was 0.81.

### Statistical analysis of data

Data analysis was performed using SPSS 25.0 and Mplus 7.0. As in the original article, clinically significant impairment/distress was not included in the psychometric properties and factor structure analysis [35]. Given that all the items were dichotomous, Kuder-Richardson's α (KR-20) was used to assess the internal consistency of the mYFAS 2.0. The test–retest correlation coefficient (intraclass correlation coefficient, ICC) was used to calculate the scale's stability. Values of ICC were interpreted as follows: > 0.75 was excellent, between 0.40 and 0.75 was fair to good, and < 0.40 was poor [52].

Content validity index (CVI) and Pearson’s correlation coefficients between items and total scores were used to evaluate the content validity of the scale. The CVI including item-level content validity index (I-CVI) and average S-CVI (S-CVI/Ave) [53]. Each expert made a choice on the relevance of each item to the corresponding dimension. A 4-point rating scale was used to calculate CVI (1 = no relevance, 2 = low relevance, 3 = strong relevance, 4 = very strong relevance). EFA and CFA were used to examine the construct validity of the mYFAS 2.0. The data was randomly divided into two samples. Sample 1 consisted of 541 undergraduates (79.0% female, mean age = 20.27 years, SD = 1.30, mean BMI = 21.82, SD = 4.59), while Sample 2 consisted of 558 undergraduates (79.0% female, mean age = 20.26 years, SD = 1.45, mean BMI = 21.79, SD = 4.61). The factor ability of the correlation matrix was assessed with the Kaiser–Meyer–Olkin (KMO) statistic and Bartlett's test for sphericity [54], and EFA was conducted on Sample 1. A scree plot was used to determine the number of factors. CFA was performed on Sample 2, and the test level was α = 0.05. To assess the quality of the factor model, the following indices were estimated: minimum function chi-square (χ^2^), comparative fit index (CFI), Tucker-Lewis index (TLI), standardized root mean residual (SRMR), and the root mean square of approximation (RMSEA). An acceptable model should have a χ^2^/*df* < 3, a RMSEA and a SRMR < 0.08 [55], and a CFI and a TLI > 0.9 [56]. The correlation between the mYFAS 2.0 symptom count score and BMI, binge episodes, self-esteem and impulsivity was evaluated by calculating the Pearson’s r correlation coefficient. Independent sample t-tests or single-factor ANOVA of the difference in the total score of symptom counts between sociodemographic classification and clinical variables and Bonferroni’s test were used to calibrate the inspection level for pairwise comparisons. Then, the total symptom count score was taken as the dependent variable, and the classified and continuous variables were used as independent variables for multivariate linear regression analysis. According to the requirements of multivariate linear regression for independent variables, the multi-classified disordered variables were set to dumb variables. The significance level was considered at *p* < 0.05.

## Results

### Descriptive statistics

In our study, most subjects were women (79.9%, n = 878). The mean age of the participants was 20.26 ± 1.38 years (age range: 18–26 years), and the mean BMI was 21.74 ± 4.24 kg/m^2^. Based on the cut-off values of BMI for Chinese adults [50], there were 231 (21.0%) underweight participants, 643 (58.5%) normal weight participants, 129 (11.7%) overweight participants, and 96 (8.7%) obese participants. Other demographic characteristics of the study sample are presented in Table 1.

**Table 1:** Sample characteristics

Characteristics	Total (N = 1099) N (%)/M ± SD
Age (years)	20.27 ± 1.38
*Sex*	
Male	221(20.1)
Female	878(79.9)
*Grade*	
Freshman	70(6.4)
Sophomore	750(68.2)
Junior	130(11.8)
Senior	149(13.6)
BMI (kg/m^2^)	21.74 ± 4.24
*Dieting ideas for weight loss*	
Had dieted or are dieting to lose weight	70(6.4)
Had dieting ideas but did not implement	750(68.2)
Did not have dieting ideas	130(11.8)
Resisted dieting	149(13.6)
*Smoking*	
No	1035(94.2)
Yes, less than once a day	27(2.5)
Yes, every day	37(3.4)
*Drinking*	
No	819(74.5)
Yes, less than once a day	256(23.3)
Yes, every day	24(2.2)
Desire to overeat (per week)	1.81 ± 2.27
Self-Esteem (SES) score	28.96 ± 4.52
Barratt Impulsiveness (BIS-8) score	17.57 ± 3.50

### Reliability analysis

The Chinese mYFAS 2.0 scale consists of 11 items (without the items for clinical significance). The item analyses (means, standard deviations, and correlations with the scale) are presented in Table 2. The KR-20 of the mYFAS 2.0 was 0.840. As seen in Table 2, the overall internal consistency of the scale was not improved by deleting any item. After two weeks, the test–retest ICC of the Chinese version of the mYFAS 2.0 was 0.857, which met the recommended criteria [52], representing good stability.

**Table 2:** Diagnostic criteria and descriptive characteristics of the Chinese mYFAS 2.0 scale items

Items	Met Criteria	Did Not Meet Criteria	Mean	SD	Correlation with the Scale	Alpha when item dropped
Substance taken in larger amount and for longer period than intended	193 (17.6%)	906 (82.4%)	0.18	0.38	0.62	0.83
Persistent desire or repeated unsuccessful attempts to quit	96 (8.7%)	1003 (91.3%)	0.09	0.28	0.53	0.83
Much time/activity to obtain, use, recover	214 (19.5%)	885 (80.5%)	0.19	0.40	0.54	0.84
Important social, occupational or recreational activities given up or reduced	167 (15.2%)	932 (84.8%)	0.15	0.36	0.67	0.82
Use continues despite knowledge of adverse consequences	105 (9.6%)	994 (90.4%)	0.10	0.29	0.64	0.82
Tolerance	75 (6.8%)	1024 (93.2%)	0.07	0.25	0.48	0.84
Characteristic withdrawal symptoms: substance taken to relieve withdrawal	265 (24.1%)	834 (75.9%)	0.24	0.43	0.63	0.83
Continued use despite social or interpersonal problems	240 (21.8%)	859 (78.2%)	0.22	0.41	0.68	0.82
Failure to fulfil major role obligations	121 (11.0%)	978 (89.0%)	0.11	0.31	0.69	0.82
Use in physically hazardous situations	146 (13.3%)	953 (86.7%)	0.13	0.34	0.69	0.82
Craving, or a strong desire or urge to use	154 (14.0%)	945 (86.0%)	0.14	0.35	0.69	0.82

SD standard deviation, Correlation with the scale = item-to-total correlations

### Construct validity analysis and model comparison

**Fig. 1:**
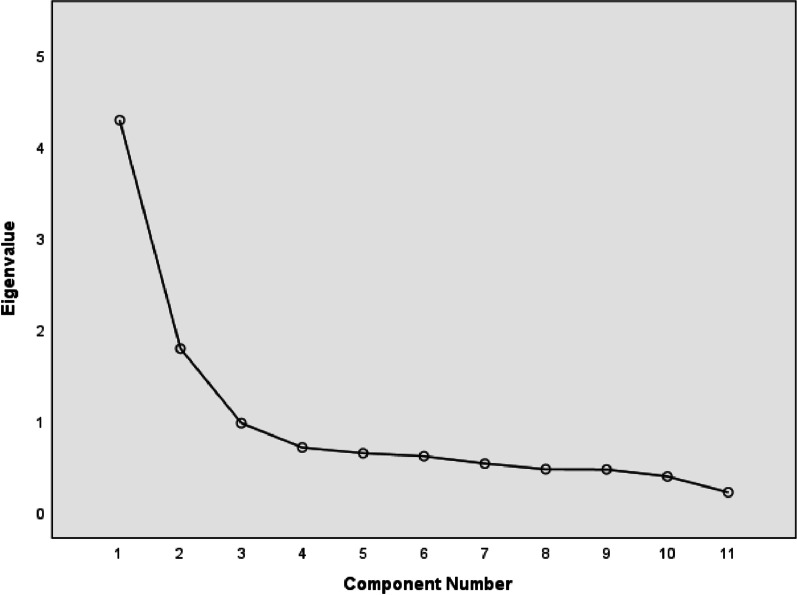
Screen plot of exploratory factor analysis for the Chinese version of the mYFAS 2.0

The statistically significant results of Bartlett's test of sphericity (χ^2^(55) = 2173.4, *p* < 0.001) and the Kaiser–Meyer–Olkin Measure of Sampling Adequacy > 0.80 (KMO = 0.861) indicate that the data meet the conditions for using factor analysis. Therefore, the first principal components analysis (PCA) was run to determine the likely number of factors. As a result, two factors that explained a total of 55.142% of the variance had initial eigenvalues > 1 each. The scree plot further confirmed the two-factor structure. After varimax orthogonal rotation, these two extracted factors explained 38.944% and 16.198% of the variance. The scree plot is shown in Fig. 1. Table 3 presents the factor loading of each item. All the correlation coefficients were more prominent than 0.50 and statistically significant at *P* < 0.01. According to the characteristics and meanings of each factor, combined with the diagnostic criteria of SUD in the DSM-5 chapter on substances and related addiction disorders [33]. Examination of the meaning in the items of the two factors revealed additional dimensions of the mYFAS 2.0 in China. After discussion by the research team, it was decided to name these two dimensions “behavioural symptoms of food addiction” and “adverse consequences of food addiction”.

**Table 3:** Factor loadings of the Chinese mYFAS 2.0 (n = 541; salient factor loadings are indicated in italics.)

	Items	Factor 1	Factor 2
Q1	I ate to the point where I felt physically ill	*0.710*	0.144
Q2	I spent more time feeling sluggish or tired from overeating	*0.687*	−0.017
Q4	If I had emotional problems because I had not eaten certain foods, I would eat those foods to feel better	*0.666*	0.217
Q11	I tried and failed to cut down on or stop eating certain foods	*0.640*	0.014
Q10	I had such strong urges to eat certain foods that I could not think of anything else	*0.636*	0.366
Q8	I kept eating in the same way even though my eating caused emotional problems	*0.618*	0.351
Q9	Eating the same amount of food did not give me as much enjoyment as it used to	*0.514*	0.120
Q7	My overeating got in the way of me taking care of my family or doing household chores	0.065	*0.898*
Q12	I was so distracted by eating that I could have been hurt (e.g., when driving a car, crossing the street and operating machinery)	0.108	*0.885*
Q3	I avoided work, school or social activities because I was afraid I would overeat there	0.159	*0.775*
Q13	My friends or family were worried about how much I overate	0.300	*0.723*

**Fig. 2:**
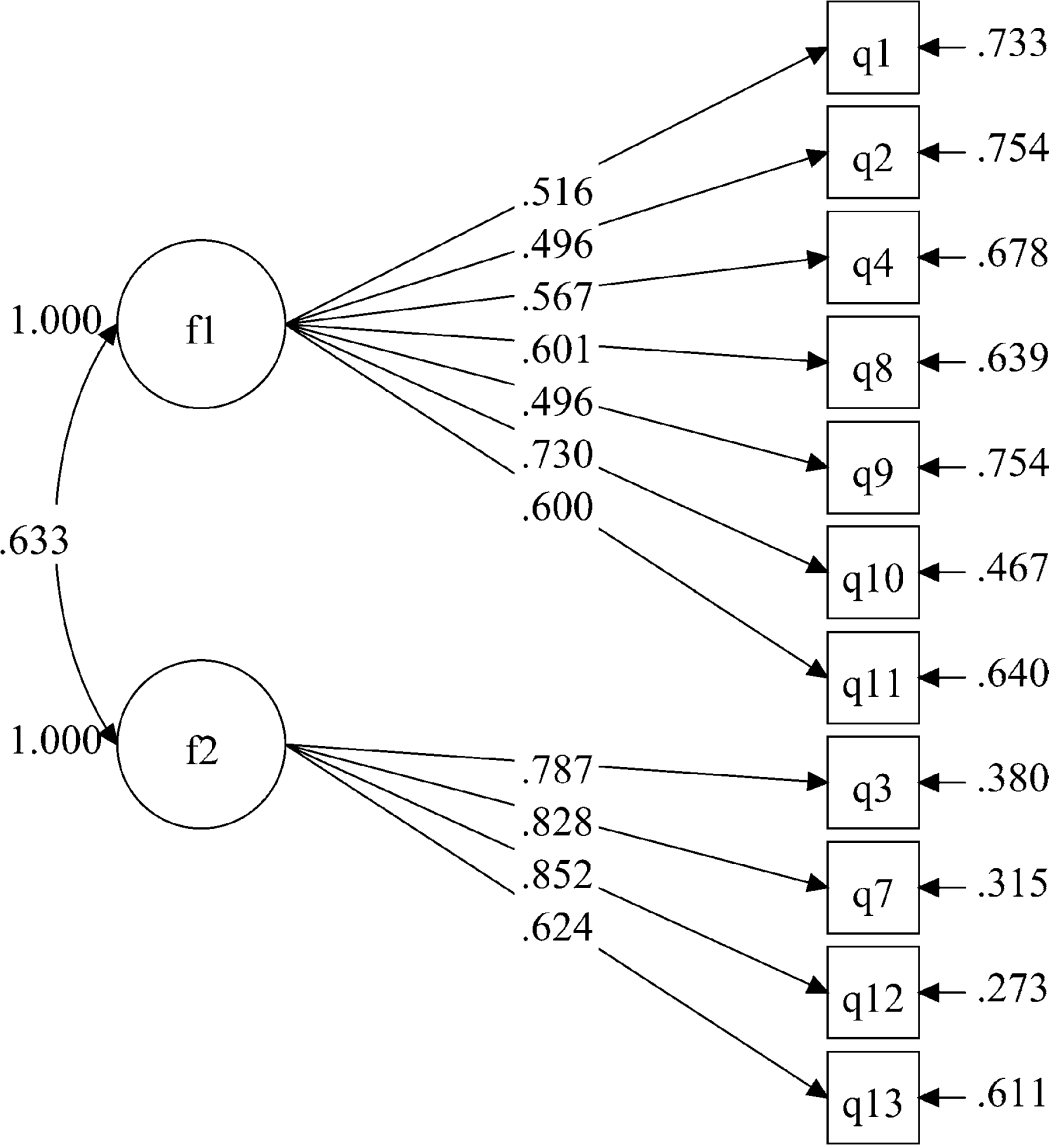
Standardized two-factor structural model of the Chinese version of the mYFAS 2.0 (n = 558); F1 (behavioural symptoms of food addiction, 7 items), F2 (adverse consequences of food addiction, 4 items)

A CFA was performed on Sample 2 (n = 558). The single-factor model of the original scale and the two-factor model of this study were evaluated, and the results showed that the fitting index of the two-factor model was better than that of the single-factor model (Table 4). The structural equation model and the standardized regression coefficients of the two-factor model of the mYFAS 2.0 appear in Fig. 2.

**Table 4:** Confirmatory factor analysis of the Chinese mYFAS 2.0 scale with different factor structures

Model	χ^2^	*df*	χ^2^/*df*	CFI	TLI	SRMR	RMSEA [90% CI]
M1	425.365	44	9.667	0.812	0.765	0.081	0.125[0.114–0.136]
M2	126.108	43	2.933	0.959	0.948	0.042	0.059[0.047–0.071]

χ2, chi-square; df, degrees of freedom; CFI, comparative fit index; TLI, Tucker-Lewis index; SRMR, standardized root mean residual; RMSEA, root mean square error of approximation; 90% CI, 90% confidence interval; M1, one-factor structure model; M2, two-factor model

### Discriminant validity

Through CFA to compare the degree of fit of different models, the results are shown in Table 4. The overall fit of the two-factor model used in this study is good, and the fitting effect is better than the single-factor model, indicating that the variables have good discriminative validity [57].

### Content validity

The item-to-total correlations ranged between 0.48 and 0.69 (which are shown in Table 2), the difference was statistically significant. The content validity of the Chinese version of mYFAS 2.0 was evaluated by expert evaluation [58]. The expert group is composed of 7 experts, including 3 psychology experts, 2 nursing experts proficient in both Chinese and English, and 2 psychiatrists. The content validity analysis result shows that the I-CVI of mYFAS 2.0 is 0.860–1.000, and the S-CVI / Ave is 0.946, which has good content validity.

### Food addiction prevalence and sociodemographic differences

The Chinese mYFAS 2.0-diagnosed food addiction symptom count was 1.616 (SD = 2.388; range = 0–11). The proportions of the subjects who met the threshold for each diagnostic criterion ranged from 6.8 to 24.1% (Table 2). A total of 74 (6.7%) subjects were regarded as having food addiction: 13 (1.2%) received a mild diagnosis, 17 (1.5%) a moderate diagnosis, and 44 (4.0%) received a severe food addiction diagnosis using the Chinese mYFAS 2.0.

There were no significant differences in the mYFAS 2.0 scores between men and women or between grade levels. However, there were statistically significant differences in dieting ideas for weight loss, alcohol use, and tobacco use. The specific results are shown in Table 5. Table 6 presents the factors associated with the mYFAS 2.0 symptom score: the mYFAS 2.0 symptom score was positively correlated with BMI, the frequency of desires to overeat per week, and impulsivity and negatively correlated with self-esteem. The effect of different sociodemographic groups on the Chinese mYFAS 2.0 scale score was assessed by linear regression. The sociodemographic groups were considered categorical predictors (with one category being the reference group), and the Chinese mYFAS 2.0 score represented the continuous outcome variable.

**Table 5:** Comparison of the Chinese mYFAS 2.0 scores of subjects with different characteristics

	M	SD	t|F	*p*-value	Pairwise differences^a^
*Sex*			0.819	0.366	
1. male	1.66	2.49			
2. female	1.61	2.36			
*Grade*			0.595	0.619	
Freshman	1.37	2.55			
Sophomore	1.59	2.36			
Junior	1.66	2.41			
Senior	1.81	2.42			
*Dieting ideas for weight loss*			7.219	**0.000**	
Had dieted or are dieting to lose weight (1)	1.98	2.58			(1) > (3), (4)
Had dieting ideas but did not implement (2)	1.61	2.40			
Did not have dieting ideas (3)	1.27	2.22			
Resisted dieting (4)	0.95	1.47			
*Smoking*			4.361	**0.013**	
No (1)	1.56	2.35			(1) < (3)
Yes, less than once a day (2)	2.44	3.13			
Yes, every day (3)	2.49	2.57			
*Drinking*			10.763	**0.000**	
No (1)	1.52	2.30			(1), (2) < (3)
Yes, less than once a day (2)	1.79	2.51			
Yes, every day (3)	3.21	3.22			

M, mean; SD, standard deviation; t, t-test; F, analysis of variance; Bold values correspond to statistically significant correlations (*p* < 0.05); Pairwise^a^ differences were *p* < 0.05 (Bonferroni corrected)

**Table 6:** Pearson's correlations between the mYFAS 2.0 symptom count and BMI, desire to overeat, self-esteem and impulsivity

	1	2	3	4	5
1. mYFAS 2.0 symptom count	–				
2. BMI	0.180**	–			
3. Desire to overeat	0.343**	0.043	–		
4. Self-Esteem	−0.263**	−0.043	−0.137**	–	
5. Impulsiveness	0.268**	0.045*	0.190**	−0.551**	–

mYFAS 2.0: modified Yale Food Addiction Scale 2.0; BMI, body mass index; Self-Esteem: the Self-Esteem Scale; Impulsiveness: the Barratt Impulsiveness Scale Brief Version; the desire to overeat: the frequency of desires to overeat per week; ** *p* < 0.01; * *p* < 0.05

On the basis of the multivariate regression analysis results shown in Table 7, the mYFAS2.0 symptom count score increased by 0.081 points for each one-unit increase in BMI and 0.302 points for each one-unit increase in the number of binge-eating episodes per week. In terms of dieting ideas for weight loss, the Chinese mYFAS 2.0 symptom count scores of the respondents who resisted dieting and the group that did not have dieting ideas were significantly lower than the Chinese mYFAS 2.0 symptom count scores of the group who had dieted or were dieting to lose weight, and the results were statistically significant. There were no significant differences in the mYFAS 2.0 symptom count scores between the tobacco use groups or the alcohol use groups. The degrees of self-esteem and impulsivity were correlated with the symptom count scores. With every increase of one SD in the self-esteem scale, the Chinese mYFAS 2.0 score decreased by 0.145 SD. 0.117 SD increased the score of the Chinese mYFAS 2.0 for each increase of one SD in the BIS-8 scale.

**Table 7:** Results of multiple linear regression models of factors influencing the Chinese mYFAS 2.0 scores of subjects with different characteristics

	B	Std. Error	Beta	t	*P*
*Dieting ideas for weight loss*					
Had dieted or are dieting to lose weight (1)	Reference				
Had dieting ideas but did not implement (2)	−0.348	0.156	−0.068	−2.237	**0.026**
Did not have dieting ideas (3)	−0.378	0.172	−0.067	−2.194	**0.028**
Resisted dieting (4)	−0.643	0.259	−0.072	−2.484	**0.013**
*Smoking*					
No (1)	Reference				
Yes, less than once a day (2)	0.405	0.428	0.026	0.947	0.344
Yes, every day (3)	0.427	0.395	0.032	1.082	0.280
*Drinking*					
No (1)	Reference				
Yes, less than once a day (2)	0.111	0.159	0.020	0.697	0.486
Yes, every day (3)	0.834	0.489	0.051	1.706	0.088
*BMI*	0.081	0.015	0.144	5.275	**0.000**
*Desire to overeat (per week)*	0.302	0.029	0.287	10.402	**0.000**
*Self-Esteem*	−0.077	0.017	−0.145	−4.474	**0.000**
*Impulsiveness*	0.080	0.022	0.117	3.567	**0.000**

BMI, body mass index; Self-Esteem: the Self-Esteem Scale; Impulsiveness: the Barratt Impulsiveness Scale Brief Version; the desire to overeat: the frequency of desires to overeat per week; *P*, *P*-Value; Bold values correspond to statistically significant correlations (*p* < 0.05)

## Discussion

This study shows that the Chinese mYFAS 2.0 scale has a two-factor structural solution and has good psychometric characteristics. The reliability analysis results of this study showed that the internal consistency coefficient of the scale meets the statistical requirements, and the Chinese mYFAS 2.0 test–retest reliability was good, indicating that the scale has good stability over time. Furthermore, the Chinese mYFAS 2.0 has good construct validity, discriminant validity and content validity.

A two-factor structure of the mYFAS 2.0 was confirmed as the best solution for the scale through CFA, which is different from the single-dimensional theoretical conception of the original scale [35, 40]. First, during the translation process, items that did not conform to Chinese expression habits were adjusted and cross-culturally adjusted, which affected the original structure of the scale to a certain extent. Second, this difference may be related to the sample population and region. Our survey samples were mainly in Northeast China. Therefore, people in different regions may have different subjective experiences of food addiction and understandings of the concept of food addiction. Other possible reasons for the difference are the different living habits, eating habits, and cultures. China has a vast territory and different dietary compositions. Our samples were mainly in Northeast China, where the diet is high in salt, sugar, and fat [59]. A survey of geographic differences in the prevalence of obesity in Chinese adults shows that Liaoning province was part of the high-prevalence cluster for general obesity in both sexes [60]. The dietary differences of the sample population may be an important reason for the different results.

From the content point of view, there are relatively reasonable explanations for various factors. The chapter on substances and related addiction disorders in the DSM-5 notes that the 11 diagnostic criteria for SUD can be divided into four groups, which are applicable to the symptoms of control damage, social damage, use risk, and pharmacological criteria [33]. Social damage, the second group of diagnostic criteria, corresponds to the three items in factor 2 of this study: affecting personal work, school, and social interaction (Item 3), affecting family care and housework (Item 7), and causing social or interpersonal problems (Item 13). The third group of substance use risks in SUD includes two criteria, one of which, the physical damage criterion, which corresponds to the use in physically hazardous situations (Item 12) in this study. The four items (Items 3, 7, 12, 13) of the mYFAS 2.0 in Dimension 2 represent the damage caused by food addiction, that is, the adverse consequences caused by food addictive. Another diagnostic criterion of substance use risk is that although the individual is aware of the physical or psychological problems that may be caused, the individual continues to use the substance. This criterion corresponds to Item 8 in the mYFAS 2.0 and belongs to factor 1 in this study. The connotations of the seven items in Dimension 1 (Items 1, 2, 4, 8, 9, 10, 11, which are shown in Table 3) are the performance of food addiction in daily life, and they are all behaviour-related symptoms. Based on the analysis of the item's specific content and factor connotation, the research team believes that dividing it into two factors is more in line with the Chinese cultural background, and the two factors are named "behavioural symptoms of food addiction" and "adverse consequences of food addiction."

The mYFAS 2.0-diagnosed food addiction prevalence was 6.7% in our subjects. Similar findings were reported using the mYFAS 2.0 with Italian undergraduate students (5.7%) [37] and France's non-clinical population (6.4%) [39]. In addition, studies using the mYFAS in large samples from six Asian countries/regions (6.2%) [61] and a previous study using the original version of the YFAS in normal-schools in China (6.91%) [62] also reported similar prevalence rates. The characteristics of the subjects in these studies have certain similarities, which may explain the similar prevalence.

In this study, BMI showed small, positive associations with the mYFAS 2.0 symptom scores. Similar findings have been found in many studies. For example, in the original scale, the correlation between the total symptom count score and BMI was 0.23 [35], and the correlation between the total symptom count score and BMI in the Italian and French YFAS 2.0 versions was 0.168 and 0.29 [63], respectively. Additionally, a previous survey using the YFAS in China showed that the correlation between the symptom count and BMI was 0.134 [62]. Compared with people without food addiction, the obesity measurements of body mass index, weight, body fat, and trunk fat of people with food addiction are higher [13]. The reason is that food-addicted individuals eat faster than non-food-addicted individuals and consume more total calories, which makes these individuals more likely to develop obesity [64]. A. Meule found that approximately 15–25% of obese individuals are food addicts [65]. Moreover, obese individuals often experience dissatisfaction with their body images, and these negative emotions may induce more serious food addiction behaviours [45].

This study shows that the idea of dieting to lose weight can have an impact on food addiction. Participants who have implemented or are implementing the idea of dieting to lose weight are more likely to be addicted to food. Dietary restriction is considered to be an essential background factor related to food addiction [66]. However, there are some inconsistencies in the relationships between food addiction, dietary restriction, and weight in existing studies. A. Meule pointed out: "The broad concept that dieting causes people to crave food has been oversimplified [67]." Some studies have shown that for chronic dieters who have been concerned about weight and weight loss for a long time, only a few achieve long-term weight loss success [68]. The survey also found that the food addiction rate of obese people who seek weight-loss treatment is significantly higher than that of obese people who are not concerned about obesity [65], and the probability reaches 30–50% [31]. Dieting is stressful [69], which may explain why engaging in dieting behaviours aimed at losing weight can actually have the opposite effect [68, 70]. In contrast, many previous studies have shown that food addiction symptoms are significantly reduced after weight loss treatment. Although short-term deprivation can increase people's cravings for avoided foods, long-term restrictions can lead to reduced food cravings [71]. Given the complexity of these findings, more research is needed to identify interventions for long-term changes in food addiction and to elucidate the associations between problem foods, food addiction, and weight.

In our study, the desire to overeat was positively correlated with the mYFAS 2.0 symptom count scores: the more frequent binge eating, the greater the possibility of food addiction. DiFeliceantonio et al. reported that the addictive response to ultra-processed foods might be a relevant contributor to bingeing [72]. Studies have shown that scores on the mYFAS 2.0 are strongly associated with binge eating episodes and binge eating symptoms [35, 37, 40]. Moreover, Brunault et al. used both the short and full YFAS 2.0 in non-clinical and clinical French-speaking samples, which confirmed that the severity of food addiction was related to more severe binge eating symptoms [39].

We found a negative correlation between the mYFAS 2.0 score and self-esteem: the lower the self-esteem scale score, the higher the summary score of the mYFAS 2.0. Self-esteem is defined as a person's attitude towards himself or herself, and it may affect quality of life and health [73]. Some previous studies have shown that self-esteem is related to eating disorders, and high self-esteem plays an essential role in preventing eating disorders and physical dissatisfaction [74]. Individuals with low self-esteem tend to have low self-control [75], and food addiction itself is an unhealthy eating state [76]. On the other hand, individuals with food addiction are more sensitive than people without food addiction when facing negative evaluations about weight, which may reduce their self-esteem levels [77].

The results of this study show that the mYFAS 2.0 score and personal impulsivity are positively correlated: the higher the impulsivity score, the higher the total score of mYFAS 2.0. Most food addiction may be caused by impulsive behaviours caused by the loss of neural signal control, environmental conditions, and psychological dependence on food [78]. Thomsen and Callesen reported the role of trait impulses in multiple diseases associated with multiple addictions [79]. The findings of Chloe Kidd suggest that trait impulsivity may contribute to food addiction in adolescents [80]. An emerging body of evidence indicates that multidimensional elements of impulsivity are unique risk factors for food addiction [81].

Several limitations should be taken into account when interpreting the findings of this study. First, the sample was conveniently obtained. There was a high proportion of female participants in our sample, which may limit the generalizability to other populations. Further investigation from the angle of sex variations could offer valuable insights. Therefore, future studies should assess the reliability and validity of the mYFAS 2.0 in other populations (such as community populations, obese populations, and populations of people with clinical eating disorders) and assess the size and characteristics of the prevalence of food addiction among different samples. Second, bias was inevitable because of the self-reporting nature of this investigation. Furthermore, we used a question to ask about the frequency of binge eating per week but did not use an effective tool to assess binge eating, which may limit the comparison of our results with others.

## Conclusion

The Chinese version of the mYFAS 2.0, supporting a two-factor structure, turned out to be reliable; therefore, it can be used as a short method of food addiction screening. Food addiction is associated with BMI, the idea of dieting to lose weight, the desire to overeat, self-esteem, and impulsivity. Future research should be encouraged to examine the psychometric properties of this translated mYFAS 2.0 across different groups in China. In addition, the potential predictors of food addiction should be further determined.

## Supplementary Information

**Additional file 1: Appendix 1.** The Chinese version of the modified Yale Food Addiction Scale 2.0

**Additional file 2: Appendix 2.** The scoring of the modified Yale Food Addiction Scale 2.0

## Data Availability

The data set is available from the corresponding author upon reasonable request.

## Abbreviations

mYFAS 2.0: The Modified Yale Food Addiction Scale Version 2.0
HP: Highly processed
BIS-8: The Barratt Impulsivity Scale
SES: The Self-Esteem Scale
BMI: Body Mass Index
PCA: Principal components analysis
SUD: Substance use disorder
ICC: Intraclass correlation coefficient
