# Supplementary material for: Translation of the Chinese version of the modified Yale Food Addiction Scale 2.0 and its validation among college students
Source: J Eat Disord. 2021 Sep 16;9:116. doi: 10.1186/s40337-021-00471-z (PMC8444594; doi:10.1186/s40337-021-00471-z)
Supplement: Supplementary file 1 — Additional file 1: Appendix 1. The Chinese version of the modified Yale Food Addiction Scale 2.0 [file 40337_2021_471_MOESM1_ESM.pdf]

Modified Yale Food Addiction Scale Version 2.0

改良版耶鲁食物成瘾量表 2.0 版

这个调查询问了你过去一年的饮食习惯。人们有时很难控制某些食物的摄入量，例如:

甜食如冰淇淋、巧克力、甜甜圈、曲奇饼干、蛋糕、糖果;

淀粉类如馒头、面包、面条和米饭;

咸味零食如薯片、辣条、饼干;

脂肪类食品如牛排、火腿、汉堡包、芝士汉堡、比萨饼和炸薯条;

含糖饮料如汽水、柠檬水、奶茶、运动饮料、还有能量饮料;

当以下问题问及某些食物时，请考虑任何与上面列表中相似的食物或饮料，或者任何其他过去一年内你难以控制自己饮食量的食物。

Items (English/Chinese)
Item 1<br>I ate to the point where I felt physically ill.<br>我吃到了感觉身体不舒服的程度。<br>Item 2<br>I spent more time feeling sluggish or tired from overeating.<br>由于吃得过多，我有很多时间感到懒散或疲倦。<br>Item 3<br>I avoided work, school or social activities because I was afraid I would overeat there.<br>我逃避工作、上学或社交活动，因为我害怕在那里会吃得过多。<br>Item 4<br>If I had emotional problems because I had not eaten certain foods,I would eat those foods to feel better.<br>如果我因为没有吃某些食物而出现情绪问题，我会吃这些食物来使自己的感觉好一些。<br>Item 5<br>My eating behaviour caused me a lot of distress.<br>我的饮食习惯给我带来了许多痛苦。<br>Item 6<br>I had significant problems in my life because of food and eating. These may have

been problems with my daily routine, work, school, friends, family or health.
因为食物和饮食，我的生活出现了严重的问题。这些可能是我的日常生活、工作、学校、朋友、家庭或健康方面的问题。

Item 7

My overeating got in the way of me taking care of my family or doing household chore.

我的暴饮暴食，妨碍了我照顾家人或做家务。

Item 8

I kept eating in the same way even though my eating caused emotional problems.

尽管我的饮食造成了情绪问题，但我一直以同样的方式进食。

Item 9

I kept eating in the same way even though my eating caused emotional problems.

吃同样量的食物不像以前那样带给我那么多的乐趣。

Item 10

I had such strong urges to eat certain foods that I could not think of anything else.

我有吃某些食物的强烈欲望，以至于我不想吃别的任何东西。

Item 11

I tried and failed to cut down on or stop eating certain foods.

我试着减少或停止吃某些食物，但没有成功。

Item 12

I was so distracted by eating that I could have been hurt (e.g. When driving a car, crossing the street and operating machinery).

我因为吃东西而分心，甚至可能会受伤（例如，开车、过马路和操作机器时）。

Item 13

My friends or family were worried about how much I overate.

我的朋友或家人担心我吃多了。
