# Supplementary material for: Translation of the Chinese version of the modified Yale Food Addiction Scale 2.0 and its validation among college students
Source: J Eat Disord. 2021 Sep 16;9:116. doi: 10.1186/s40337-021-00471-z (PMC8444594; doi:10.1186/s40337-021-00471-z)
Supplement: Supplementary file 2 — Additional file 2: Appendix 2. The scoring of the modified Yale Food Addiction Scale 2.0 [file 40337_2021_471_MOESM2_ESM.pdf]

The scoring of the modified Yale Food Addiction Scale 2.0

Items	Response options							
	Never	Less than monthly	Once a month	2-3 times a month	Once a week	2-3 times a week	4-6 times a week	Every Day
Item 1	0	1	2	3	**4**	5	6	7
Item 2	0	1	2	3	4	**5**	6	7
Item 3	0	1	**2**	3	4	5	6	7
Item 4	0	1	2	3	**4**	5	6	7
Item 5	0	1	2	3	4	**5**	6	7
Item 6	0	1	2	3	4	**5**	6	7
Item 7	0	1	**2**	3	4	5	6	7
Item 8	0	1	2	3	**4**	5	6	7
Item 9	0	1	2	3	4	**5**	6	7
Item 10	0	1	2	3	**4**	5	6	7
Item 11	0	1	2	3	4	**5**	6	7
Item 12	0	1	**2**	3	4	5	6	7
Item 13	0	1	**2**	3	4	5	6	7

Notes: Item scores are recoded to 0 and 1 (numbers that are printed in bold in this table are scored with 1). If the recoded items have a score of at least 1 within each symptom, then this symptom is met. Once a month: (=>2); Once a week: (=>4); 2-3 times a week: (=>5).
